# Lectin binding of human sperm associates with *DEFB126* mutation and serves as a potential biomarker for subfertility

**DOI:** 10.1038/srep20249

**Published:** 2016-02-01

**Authors:** Aijie Xin, Li Cheng, Hua Diao, Yancheng Wu, Shumin Zhou, Changgen Shi, Yangyang Sun, Peng Wang, Shiwei Duan, Jufen Zheng, Bin Wu, Yao Yuan, Yihua Gu, Guowu Chen, Xiaoxi Sun, Huijuan Shi, Shengce Tao, Yonglian Zhang

**Affiliations:** 1 Shanghai Ji Ai Genetics and IVF Institute, Obstetrics and Gynecology Hospital, Institute of Reproduction and Development, Fudan University, Shanghai 200011, China; 2 China National Population and Family Planning Key Laboratory of Contraceptive Drugs and Devices, SIPPR, Shanghai 200032, China; 3 Shanghai Center for Systems Biomedicine, Key Laboratory of Systems Biomedicine (Ministry of Education), Shanghai Jiao Tong University, Shanghai 200240, China; 4 Shanghai Key Laboratory of Reproductive Medicine, Shanghai Jiao Tong University School of Medicine, Shanghai 200025, China; 5 Shanghai Key Laboratory for Molecular Andrology, State Key Laboratory of Molecular Biology, Institute of Biochemistry and Cell Biology, Shanghai Institutes for Biological Sciences, Chinese Academy of Sciences, Shanghai 200031, China; 6 The Affiliated Hospital, School of Medicine, Ningbo University, Ningbo, Zhejiang 315211, China

## Abstract

Coating on the sperm surface, glycocalyx, plays a key role in sperm motility, maturation and fertilization. A comprehensive profile of sperm surface glycans will greatly facilitate both basic researches and clinical studies. Because of the capability of recognizing different glycan moieties, lectins are widely used in glycobiology. However, lacking high-throughput technology, limited lectins have been reported for analyzing the glycan of human sperm. In this study, we employed a lectin microarray for profiling the surface glycans of human sperm, on which 54 out of 91 lectins showed positive binding. Based on this technique, we compared lectin binding profiling of sperm with homozygous *DEFB126* mutation (*del*/*del*) with that of wild type (*wt*/*wt*). DEFB126 was reported to contribute to the sialylation on sperm surface and its homozygous mutation was related to male subfertility. Six lectins (Jacalin/AIA, GHA, ACL, MPL, VVL and ABA) were found to develop lower binding affinity to sperm with *del*/*del*. Further validation showed that these lectins, especially ABA and MPL, can be potential biomarkers for clinical diagnosis of subfertility due to the mutation of *DEFB126*. Our research provides insight into the detection of some unexplained male subfertility, and the lectin microarray is generally applicable for infertility/subfertility sperm biomarker discovery.

The membrane surface of mature sperm has been found to be coated with a thick layer of glycans, i.e., the sperm glycocalyx, including O- and N-glycans, which protects sperm during transit in the female reproductive tract and assists with other key functions, including attachment of sperm to oviduct epithelium, regulation of capacitation, and sperm-egg interaction^1^,^2^,^3^,^4^,^5^,^6^. The glycans play crucial role in the binding of sperm with egg^7^. The elaborate glycocalyx of sperm is a conserved feature of epididymal maturation in mammals and the surface of mammalian sperm experiences dramatic changes which are vital for sperm to keep viability and functions in the female reproductive tract^1^,^8^,^9^,^10^. However, it is still not well understood how the sperm glycocalyx contributes to male fertility. In the macaque, β-defensin 126 (DEFB126), originally named epididymis-specific protein ESP13.2, is a multifunctional glycoprotein consisting of a conserved β-defensin core and a C-terminal glycosylated peptide tail with 20 *O*-glycosylation sites linking oligosaccharides in contributing to glycocalyx formation^11^,^12^. DEFB126 is expressed and secreted by the principal cells of epididymal distal corpus and proximal cauda epithelium and the highly sialylated DEFB126 protein is recruited to the surface of sperm during transit through the epididymal duct^11^,^13^,^14^, where the protein constitutes a major part of the sperm glycocalyx^12^. DEFB126 was reported to be essential for sperm efficiently advancing into the upper female reproductive tract, protecting sperm from immune recognition by the female immune system^15^, facilitating sperm penetration of cervical mucus^16^, and mediating attachment of sperm to oviduct epithelia^17^.

It was reported that about 20% of the Chinese males in reproductive ages had two-nucleotide deletion in *DEFB126* (rs11467417) gene on both chromosomes (*del*/*del*)^8^. Homozygous *DEFB126* mutant (*del*/*del*) shows significantly poorer penetration of Hyaluronic acid (HA, a substitute of cervical mucus), thus making the married men with *DEFB126* mutation (*del*/*del*) take longer time to get their wives pregnant than those with either *DEFB126* wild type (*wt*/*wt*) or heterozygous mutation (*wt*/*del*)^8^. And interestingly, sperm with *del*/*del* showed lower *Agaricus bisporus* (ABA) lectin binding^8^. It is highly possible that the *DEFB126* mutation (*del*/*del*) associated sperm surface glycan changes may relate to the capability of fertilization. Thus, it will be extremely valuable to get the whole profile of the sperm surface glycans, and based on this, one could easily identify surface glycan difference that is clinically meaningful, e.g., difference/s between *del*/*del*, and *wt*/*del*, *wt*/*wt* of *DEFB126* in a fast and efficient method.

As a group of natural glycan binders, lectins labeled with different conjugates including enzyme, fluorescence or biotin to detect individual glycans by immunocytochemistry, immunohistochemistry or flow cytometry are the major tools to explore the composition of the sperm glycocalyx^18^. In 2005, a novel high-throughput technique-lectin microarray came into being^19^,^20^,^21^,^22^. Owing to its high speed, accuracy and sensitivity, the technique has been extensively utilized in the analysis of bacteria^23^,^24^, fungi^22^,^25^, virus^26^ and mammalian cell surface glycome^27^,^28^; therefore, it can be a promising and powerful tool to characterize the glycome on the surface of diverse cells.

Taking advantage of the lectin microarray technology, we reported the establishment of a standard and general procedure for profiling the human sperm surface glycome in a high-throughput fashion and with less than 3 h. A detailed lectin binding profile of human sperm with 91 lectins was generated. We employed the standard procedure for fast identification of lectin binding difference/s by comparing sperm samples of three different genotypes of *DEFB126* (*i.e.*, *wt*/*wt, wt*/*del* and *del*/*del*). Several candidate lectins, i.e., Jacalin/AIA, GHA, ACL, MPL, VVL and ABA, with significant binding difference were successfully identified. ABA and MPL were further validated by a variety of assays, and the results were all found to be consistent with those from the lectin microarray. Thus, the candidate lectins may serve as novel biomarkers for the diagnosis of male subfertility.

## Results

### An optimized approach for direct profiling of the sperm surface glycome

**Figure 1:**
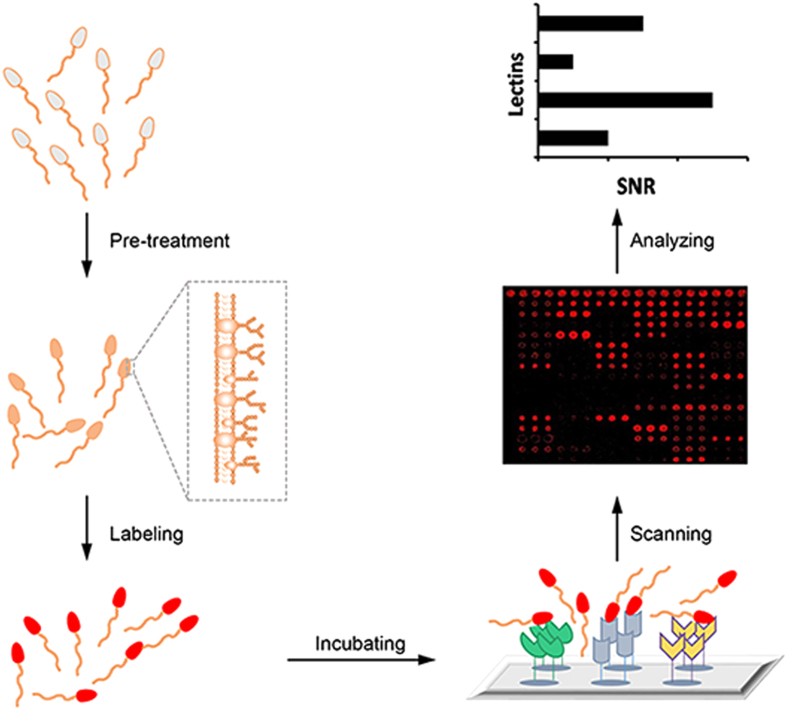
Schematic diagram for detecting human sperm surface glycans by lectin microarray. The ejaculated sperm were pre-treated and labeled with appropriate fluorescent dye, and incubated with lectin microarray. After the unbound sperm were washed off, the binding signals of sperm-lectin were then visualized, recorded and processed by a fluorescence microarray scanner coupled with appropriate software.

In order to investigate the glycocalyx of human sperm and the changes of glycans in sperm from men with *del*/*del*, we firstly optimized the approach of lectin microarray for dectecting human sperm. As shown in Fig. 1, the schematic was composed of 5 typical steps: sperm pre-treatment, labeling, incubating, scanning and data analysis.

**Figure 2:**
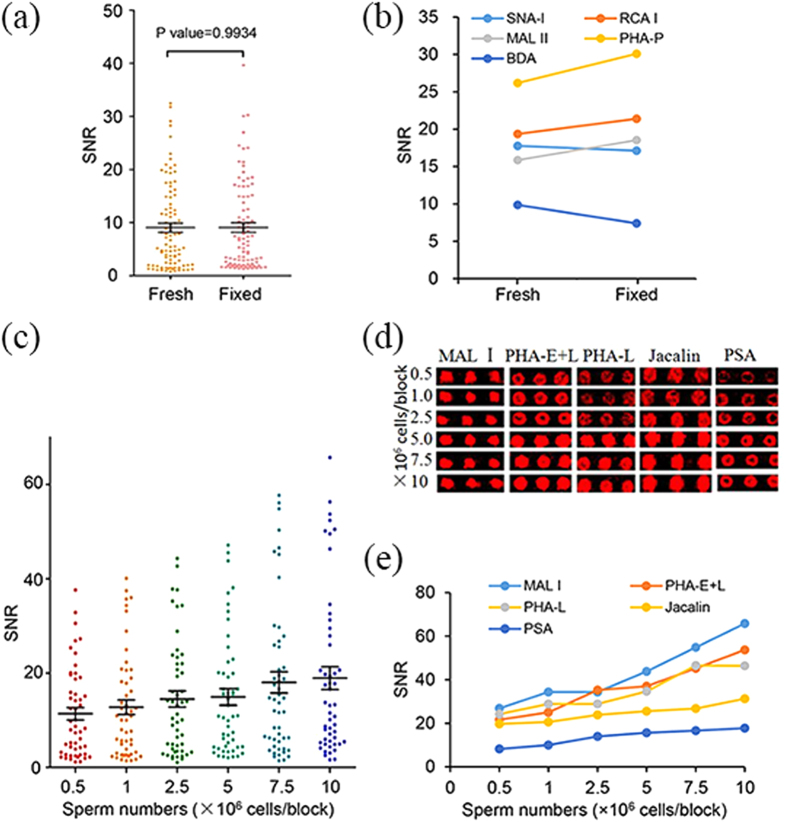
Optimization of the lectin microarray based strategy for sperm surface glycan analysis. (**a**,**b**) Effect of fixation on sperm-lectin binding; the average SNRs of the total lectins (**a**) and the five representative lectins (**b**) showing non-significant differences between the fixed sperm and the fresh ejaculated sperm; (**c–e**) the optimization of sperm concentration for lectin microarray analysis from 0.5–10 × 10^6^ sperm/block; the average SNRs of the total lectins (**c**) and the five representative lectins (**d**,**e**) depending on the sperm number.

Since sperm have different characteristics when compared with common mammalian cells, the basic steps of the lectin microarray analysis were optimized in the sample preparation. To reduce the effect of the fast motility of live sperm on sperm-lectin binding, as the binding intensity of sperm after cryopreservation was low, we compared the lectin binding patterns of the fixed sperm with the fresh samples, finding that the lectin binding patterns and fluorescence intensity were similar between them (Fig. 2a,b, *P* > 0.05).

In order to prevent the sperm-lectin binding signal saturation and save the clinic sperm samples, sperm at a range of 0.5–10 × 10^6^ sperm/block were applied to lectin microarray for optimizing sperm counts. The signal intensity to the local background noise ratio (SNR) of the total lectins (Fig. 2c; Supplementary Fig. S1) and 5 representative lectins (Fig. 2d,e) were plotted against the sperm number. The data indicated that SNRs enhanced with the increasing sperm counts (Fig. 2c,e) and the signal of some lectins like PHA-L reached saturation at 7.5 × 10^6^ cells/block, whereas most lectins located in the linear range of 2.5–7.5 × 10^6^ sperm/block (Fig. 2e). In the current study, the sperm at a concentration of 5 × 10^6^ sperm/block, which lay in the middle of the linear range, were analyzed.

Additionally, to examine whether the storage time of fixed sperm affected the lectin binding profiling, the fixed sperm stored at 4 °C from 0 day to 6 months were employed on lectin microarray. No significant difference was observed, the SNRs keeping almost the same among the samples (Supplementary Fig. S2).

### The lectin binding profiling of human sperm

The human sperm surface is decorated with a thick layer of glycans, i.e., the glycocalyx. Though it is known that some lectins have positive bindings to human sperm^10^,^29^,^30^,^31^, the overall picture of the glycan composition of human sperm glycocalyx is still unclear. We took advantage of the established lectin microarray based strategy to profile the human sperm surface glycan composition.

**Figure 3:**
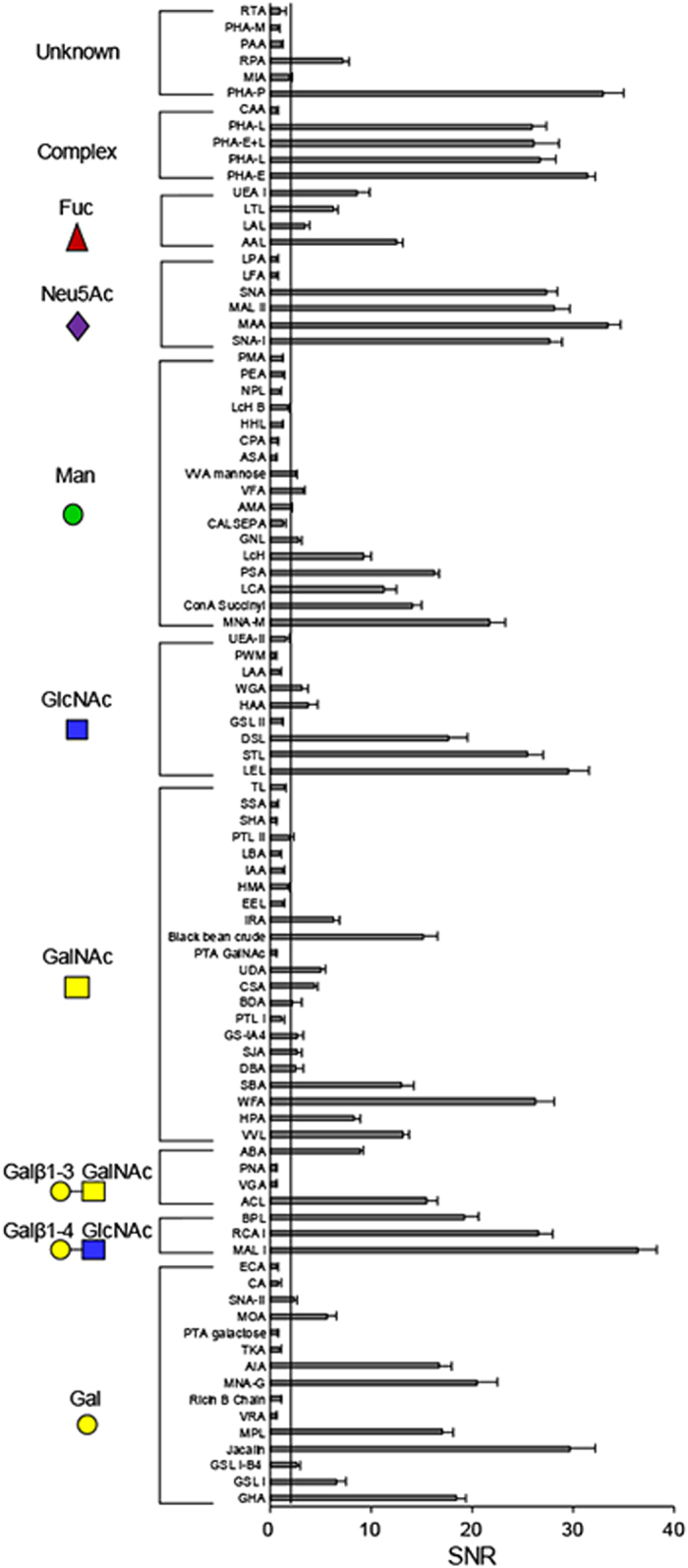
The lectin binding profiling of human sperm. The sperm-lectin binding profiling presented by the average SNR; the data being the average ± SEM of 10 samples.

To test the reproducibility, sperm samples from 10 donors with normal semen parameters and *wt*/*wt* genotype of *DEFB126* were repeatedly probed in four blocks, the sperm-lectin binding pattern of each block showing constant repeatability and almost unanimous binding patterns (Supplementary Fig. S3, R = 0.9496). As shown in Fig. 3, 91 lectins binding signal intensity of sperm were analyzed, the results showing that 54 lectins (SNR ≥ 2 being cut-off) were positive in binding sperm, which covered a wide range of glycan specificity containing galactose (Gal), N-acetylgalactosamine (GalNAc), N-acetylglucosamine (GlcNAc), mannose/glucose (Man/Glc), sialic acids (Sia), fucose (Fuc) and complex-type glycan. Of 91 lectins, many were found for the first time to be strong in the binding, such as MPL and MNA-G (Gal binders), VVL and WFA (GalNAc binders), BPL (Galβ1-3GalNAc binder), DSL and STL (GlcNAc binders), MNA-M (Man/Glc binder) and PHA-P (unknown in specificity). Additionally, α2-3-Sia (MALII and MAA) and α2-6-Sia specific lectins (SNA and SNA-I) presented strong binding intensity to the sperm. The lectin binding pattern of sperm from donors with *wt*/*wt* genotype was almost consistent with the previous report from donors with normal semen parameters^32^. The findings indicated the human sperm glycocalyx was composed of a variety of glycans.

### Expression and location of DEFB126

**Figure 4:**
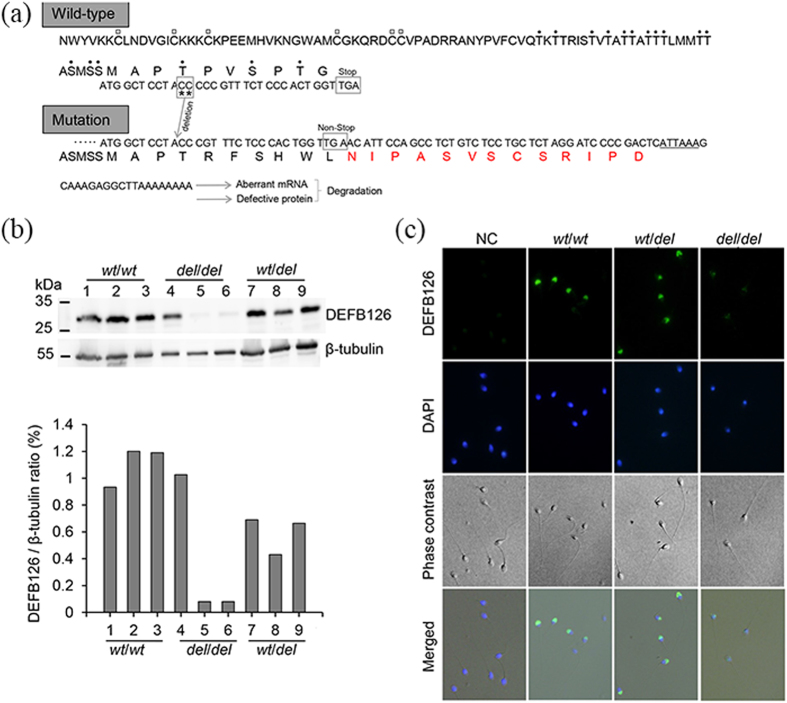
The expression and location of DEFB126 on human sperm. (**a**) The wild-type *DEFB126* and its two-nucleotide deletion mutant deduced from the mRNA nucleotide sequence; the six highly conserved cysteines (C) marked with empty squares^45^; seventeen potential residues (serine and threonine, dotted) at carboxyl terminus of DEFB126 predicted to be *O*-linked glycosylated (Net*O*Glyc 3.1)^43^; the two missed nucleotides (CC) labeled with asterisk and its frame-shifted version shown in the lower panel; the additional amino acids in the mutant protein displayed with the red front; the regulatory element of polyA signal sequence labeled with underline. (**b**) Representative Western blot from the ejaculated sperm of different genotypes (*wt*/*wt*, n = 3; *wt*/*del*, n = 3; *del*/*del*, n = 3), showing unstable expression of DEFB126 in sperm with *del/del* compared to the sperm with the other two genotypes (*wt*/*wt* or *wt*/*del*); β-tubulin used as loading control. (**c**) Localization of DEFB126 (green) on human sperm of different genotypes (*wt*/*wt*, *wt*/*del* and *del*/*del*); the sperm smears stained with polyclonal antibody against DEFB126 (rabbit anti-human β-defensin 126, sc-85535; 1:200 dilution) followed by an Alexa Fluor 488 conjugated donkey anti-rabbit IgG (1:200 dilution); sperm nuclei stained with DAPI (blue); Negative control (NC) sperm staining with rabbit IgG showing no immunoreactive staining.

Beta-defensin126 (DEFB126) has numerous potential sites (serine and threonine) for *O*-glycosylation in at the carboxyl terminus, and is a major component of the sperm glycocalyx in mouse and macaque^12^,^13^. In human, DEFB126 had 17 potential *O*-glycosylation residues predicted by Net*O*Glyc 3.1 (Fig. 4a). The reported two-nucleotide deletion of *DEFB126* would generate a non-stop mRNA and cause the aberrant mRNAs and peptides degradation by a non-stop decay (NSD) pathway and protein quality control system (Fig. 4a)^33^,^34^,^35^.

To examine whether DEFB126 could bind with human sperm, the total sperm proteins were analyzed by western blot, thus producing the results that sperm with *wt*/*wt* or *wt*/*del* genotype exhibited clear and specific bands at about 30 kDa region, larger than the theoretical mature molecular weight (10 kDa; http://www.ncbi.nlm.nih.gov/protein/NP_112193.1), while the expression of DEFB126 in sperm with *del*/*del* genotype was unstable (Fig. 4b). Some of the sperm with *del*/*del* developed a considerable amount of DEFB126, as in the case of the sperm with *wt*/*wt* or *wt*/*del*, whereas some presented very weak bands (Fig. 4b). The data of sperm immunofluorescence also showed the same results. The sperm which showed weak bands demonstrated reduced fluorescence when compared with that with *wt*/*wt* or *wt*/*del* (Fig. 4c; Supplementary Fig. S4). As not in the case of mouse and macaque^11^,^13^, DEFB126 mainly located on human sperm acrosome (Fig. 4c; Supplementary Fig. S4).

### Different surface glycan profiling of the sperm with the common mutation of *DEFB126*

To test whether *DEFB126* mutation is related to sperm surface glycan aberrance, the sperm samples from 30 donors with *wt*/*wt*, *wt*/*del* or *del*/*del* were collected and probed on the lectin microarray following the established protocols.

**Figure 5:**
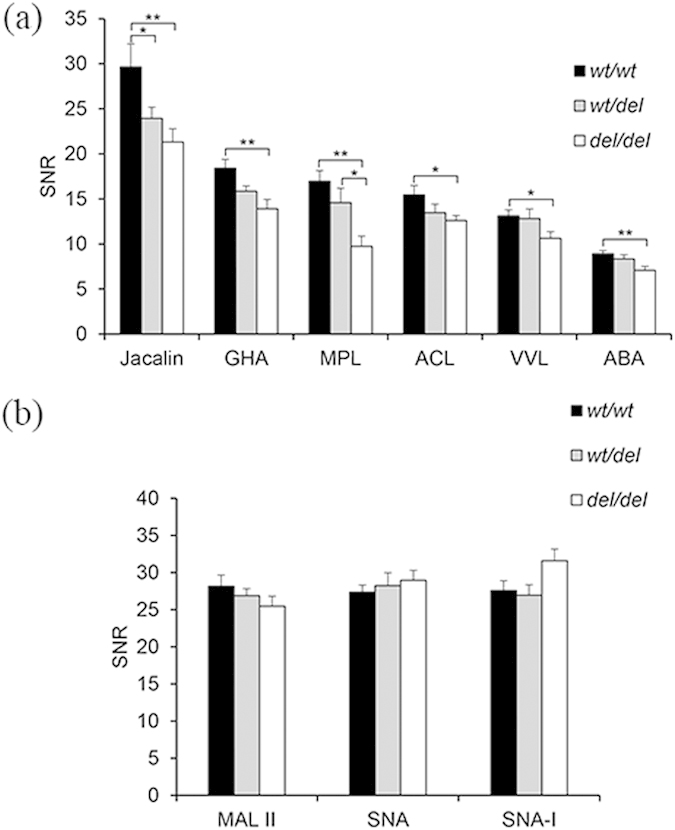
Comparison of the lectin bindings among human sperm of three genotypes, i.e., *wt/wt, wt/del* and *del/del* 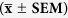; 10 samples of each genotype). (**a**) Lectins showing statistically significant sperm binding differences among human sperm with *wt*/*wt*, *wt*/*del* or *del*/*del* genotype; (**b**) Sialic acid specific lectins demonstrating similar sperm-lectin bindings among the three genotypes, *P < 0.05, **P < 0.01. Error bars showing mean values with SEM.

Upon an analysis of significant differences of lectin binding patterns by lectin microarray with software SPSS16.0, six lectins containing *Artocarpus integrifolia agglutinin* (Jacalin/AIA), *Gossypium hirsutum agglutinin* (GHA), *Amaranthus caudatus lectin* (ACL), *Maclura pomifera lectin* (MPL), *Vicia villosa lectin* (VVL) and *Agaricus bisporus agglutinin* (ABA) showed significantly reduced capacity of binding to the sperm with *del*/*del* genotype in comparison with the sperm from the wild type (Fig. 5a).

Intriguingly, the sialic acid specific lectins of MAL II (α2-3-Sia), SNA and SNA-I (α2-6-Sia) presented no significant difference in the sperm of *wt*/*wt*, *wt*/*del* and *del*/*del* in terms of binding intensity (Fig. 5b).

To rule out the differences due to the sperm heterogeneity, the semen parameters of sperm with different genotypes of *DEFB126* (*wt*/*wt*, *wt*/*del* and *del*/*del*), containing the sperm motility and viability, were analyzed and they all demonstrated no significant difference among the three groups (Supplementary Table. S1). Moreover, the binding signals of pissum sativum lectin (PSA), a well known lectin used to access acrosome reaction^36^,^37^, also showed no significant difference among the three groups (Supplementary Fig. S5).

### Validation of the different lectins binding of sperm with *DEFB126* mutation

**Figure 6:**
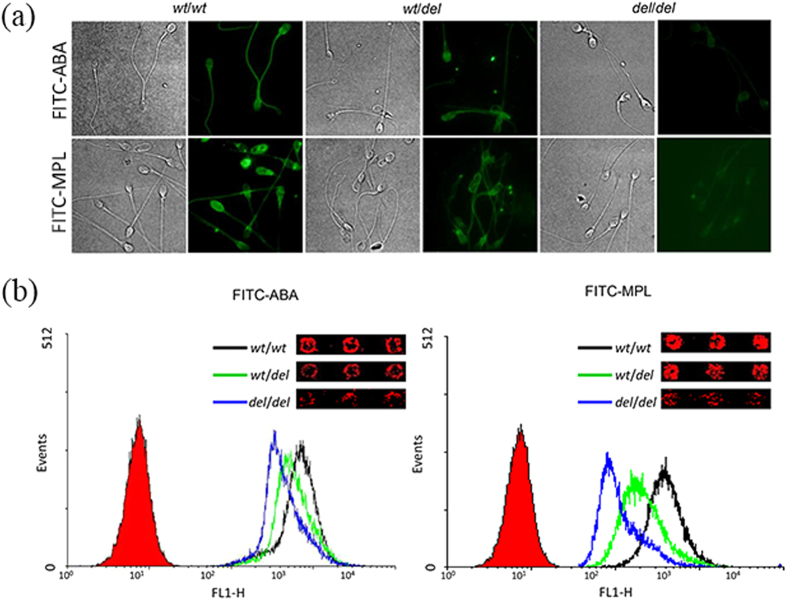
Validation of sperm-lectin binding by fluorescence microscope and FACS. (**a**) The different fluorescence signal of sperm with *wt*/*wt*, *wt*/*del* or *del*/*del* genotype labeled with FITC-ABA or FITC-MPL; the fluorescence micrographs of sperm with each genotype shown in right panel and its corresponding phase contrast shown in left panel. (**b**) FACS analysis of sperm with the three genotypes labeled with FITC-ABA or FITC-MPL; the mean channel fluorescence (MCF) of sperm with *wt*/*wt* genotype (black line) shifts being larger than those of sperm with *wt*/*del* (green line) or *del*/*del* (blue line); insets being the lectin microarray images for the sperm with respective genotypes.

To validate the different lectins binding of the sperm with *wt*/*wt, wt*/*del* and *del*/*del* genotypes, we used fluorescein isothiocyanate (FITC)-labeled ABA and MPL to analyze the binding signal of sperm by the fluorescence microscope and flow cytometry (FACS). The fluorescence signals of FITC-ABA and FITC-MPL binding sperm with *del*/*del* genotype were weaker than those of sperm with the other two genotypes (Fig. 6a). As indicated by the data from the flow cytometry, moreover, the intensity of the fluorescence was directly associated with the degree of binding capacity on the microarray (Fig. 6b).

**Figure 7:**
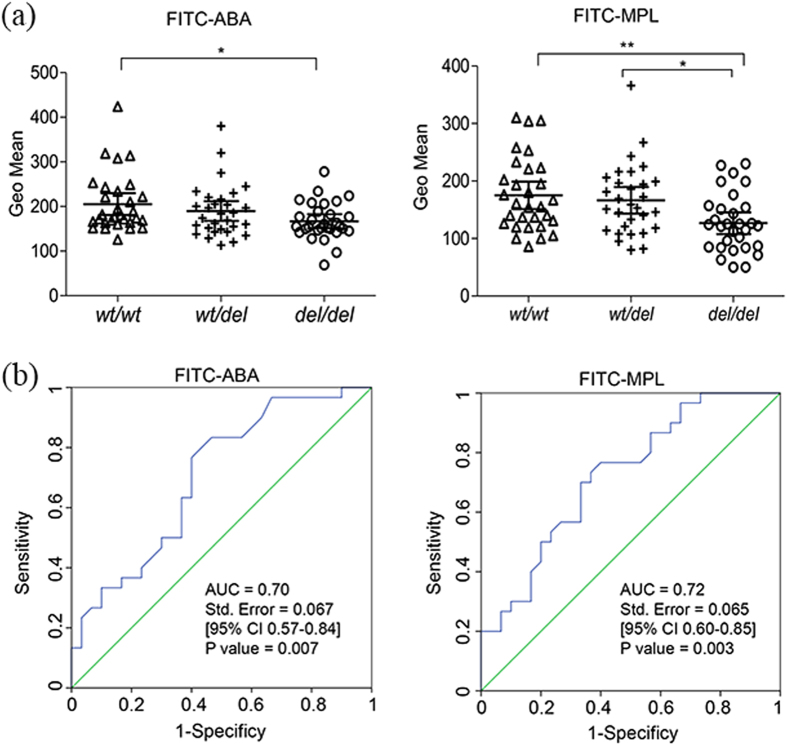
Evaluation of ABA and MPL being biomarker candidates by FACS. (**a**) The statistic difference of Lectins (ABA and MPL) verified by FACS showing complete consistency with lectin microarray; Geo Mean of lectins binding sperm with different *DEFB126* genotypes (*wt*/*wt*, n = 30; *wt*/*del*, n = 30; *del*/*del*, n = 30) analyzed by SPSS16.0. *P < 0.05, **P < 0.01. (**b**) The performance of ABA and MPL as biomarkers for assessing the sperm fertility; sperm with *wt*/*wt* genotype as the control group (n = 30) and sperm with *del*/*del* genotype as mutation group (n = 30). The ROC curves and the corresponding AUCs were calculated by SPSS16.0.

To further test the capability of the identified lectins as potential biomarkers for diagnosing subfertility due to the mutation of *DEFB126*, the fluorescence geometric mean (Geo mean) of FITC-ABA and FITC-MPL was measured among 90 donors (*wt*/*wt*, n = 30; *wt*/*del*, n = 30; *del*/*del*, n = 30) and the statistic difference between them showed high consistency with the results obtained by lectin microarray (Fig. 7a). In addition, no significant association was observed between the different *DEFB126* genotypes of the sperm and the semen parameters by the computer-assisted sperm analysis (CASA) (Table 1). Statistical analysis with receiver operating characteristic (ROC) curve demonstrated that the area under the curves (AUC) of ABA and MPL was 0.70 ± 0.067 (95% CI: 0.57–0.84, *P* < 0.01) and 0.72 ± 0.065 (95% CI: 0.60–0.85, *P* < 0.01), respectively (Fig. 7b). Such results indicated that ABA and MPL could serve as potential biomarkers for clinical diagnosis of the impaired glycocalyx due to the homozygous mutation of *DEFB126*. The cut-off values of ABA and MPL based on these data were 157.5 with 83.3% specificity (95% CI: 0.65–0.94) and 53.3% sensitivity (95% CI: 0.34–0.72), and 125.5 with 76.7% specificity (95% CI: 0.58–0.90) and 60.0% sensitivity (95% CI: 0.41–0.77), respectively.

## Discussion

Sperm glycocalyx plays an important role in sperm motility, maturation and fertilization^2^,^9^. However, the glycan profiling of sperm glycocalyx is largely unknown because of the lacking of powerful technology. In the current study, therefore, we pioneered in optimizing the procedures of lectin microarray for the global profiling of sperm surface glycans. DEFB126, a major carrier of glycocalyx carbohydrates in macaque and mouse, has been reported to be a common mutation related to sperm subfertility^8^,^12^,^13^. With the sperm of different *DEFB126* genotypes (*wt*/*wt*, *wt*/*del*, and *del*/*del*) compared, six lectins (Jacalin/AIA, GHA, ACL, MPL, VVL and ABA) displayed statistically significant differences, and the sperm with *del*/*del* showed the lower lectin binding ability. Further validation with flow cytometry showed that these lectins, especially ABA and MPL, could be biomarkers for clinical diagnosis/identification of unexplained subfertile sperm due to the mutation of *DEFB126*.

It is well recognized that DEFB126 is synthesized and secreted in the epithelial cells of epididymal corpus, and then added to the sperm surface during epididymal maturation in mouse, rat and macaque^11^,^13^,^14^. The protein has a long glycosylated carboxyl peptide tail contributing substantially to the sperm glycocalyx of nonhuman primates^9^,^12^. In human, our results of western blot and immunofluorescence indicated that DEFB126 also existed on the human sperm. In addition, DEFB126 had 17 possible glycosylation sites for glycosylation (Fig. 4a). As indicated by the specific bands, the protein at 30 kDa was greater than 10 kDa of its theoretical molecular weight (Fig. 4b), which might be ascribed to protein glycosylation. The two-nucleotide deletion in *DEFB126* gene on both chromosomes (*del*/*del*) has been reported to result in a reading frame shift and generate a nonstop mRNA prone to degradation due to NSD surveillance mechanism^8^,^33^,^34^. Additionally, the level of protein product of nonstop mRNA containing a poly (A) might be reduced because of translation repression and protein destabilization by proteasome^35^. We found that the protein DEFB126 of sperm with *del*/*del* genotype was indeed unstable (Fig. 4b).

As a sensitive and high-throughput technology, lectin microarray has already been widely employed in profiling the surface glycans of a variety of human cells^27^,^28^. We applied the technology to exploring human sperm, and optimized its procedures such as fixation, staining, sperm concentration, etc. Specifically, a fixation step with the surface glycans well preserved was established for application after the sperm samples were collected. After fixation, the sperm could be stably stored at 4 °C for at least 6 months (Supplementary Fig. S2). The fixation not only made sperm lose motility, thus facilitating binding lectin microarray, but also provided the possibility of comparing the sperm samples collected at different time points in a consistent and reliable way.

Sperm are the most diverse cell type known. Different semen samples, even from the same individual at different time point, show highly variable characteristics, especially the sperm morphology, motility, vitality, and spontaneous acrosome reaction rate^38^. The mobile sperm enriched by centrifugation on percoll gradient presented enhanced fluorescent lectin binding^39^. It will be interesting to examine the relationship between the sperm surface glycosylation and these heterogeneous characteristics. And the lectin microarray is a suitable tool to compare the lectin binding profiling among the heterogeneous sperm. However, in the current study, the semen parameters, containing the sperm motility and viability, demonstrated no significant difference among sperm with different genotypes of *DEFB126* (*wt*/*wt*, *wt*/*del* and *del*/*del*; Supplementary Table S1). On the other hand, it is reported that the spontaneous acrosome reaction rate in human spermatozoa is generally less than 15% under physiological conditions^40^. The binding signal of pissum sativum lectin (PSA), a well known lectin used to access acrosome reaction^36^,^37^, to the three groups (*wt*/*wt*, *wt*/*del* and *del*/*del*) also showed no significant difference (Supplementary Fig. S5). Nevertheless, to reduce the difference among individuals, 10 samples were analyzed on the lectin microarrays (Fig. 3), only the averaged data with standard deviations were presented.

We compared sperm with three genotypes of *DEFB126, i.e., wt*/*wt, wt*/*del* or *del*/*del* by the lectin microarray, finding that 6 lectins of Jacalin/AIA, GHA, ACL, MPL, VVL and ABA displayed significantly reduced binding ability in the sperm of *del*/*del* homozygotes (Fig. 5a). Of these lectins, ABA and MPL were further validated; the results were observed to be well consistent with those of lectin microarray (Fig. 6). The lower binding ability of ABA on sperm with *del*/*del* genotype was similar as described previously^8^, which further verified the reliability of the lectin microarray for exploring sperm surface glycome. Additionally, the 6 lectins recognized specifically β-galactose and/or N-acetylgalactosamine oligosaccharide. Especially, ABA and Jacalin lectins recognized the site specific for *O*-linked glycosylation (galactose-N-acetylgalactosamine-serine/threonine)^12^. The carboxyl tail of human DEFB126 has 17 predicted *O*-linked glycosylation sites, which suggested that the lower binding of those 6 lectins to sperm of *del*/*del* may be ascribed to the lower abundance of *O*-glycosylation of DEFB126, i.e., GalNAc residues. This demonstrated that the post-translational glycosylation of DEFB126 in human might be consistent with that in the previously reported macaque^12^. However, the Jacalin/ABA binding intensity was still high in sperm with *del*/*del* genotype, which may be ascribed to the compensation of the other glycans or glycosylated proteins in *del*/*del* sperm.

Sialic acids, the outmost glycocalyx of sperm, contribute mainly to the negative charge of sperm^41^. DEFB126 is endowed with negative charges due to its possession of sialic acid; treatment of DEFB126 with neuraminidase releases sialic acid and renders DEFB126 neutrality^12^. It is the negative charges on DEFB126 on the sperm surface that allow sperm to move through the cervical mucus, which is enriched in anionic glycosaminoglycans^9^. Upon capacitation, DEFB126 is released from the sperm surface^9^,^42^. If *del*/*del* mutation results the loss of DEFB126, the level of sialic acid has to be reduced. In the current study, however, the sialic acid specific lectins (MALII, SNA and SNA-I) demonstrated an almost invariable binding intensity with the sperm of the three genotypes via lectin microarray (Fig. 5b). This suggested that the reduced cervical mucus penetration ability of sperm with *del*/*del* was not caused by the loss of negative charge results from the lessened sialic acids. So far, there has been no definite proof that sperm glycocalyx is equivalent to the glycans of DEFB126. Free glycans and glycans of other glycoproteins can be part of sperm glycocalyx^2^. Compensation of the level of sialic acid can occur on the other sperm surface glycoproteins or glycans. In addition, the glycan structures of human DEFB126 have not been reported yet. However, if native DEFB126 could be obtained with a fair amount from human sperm, the glycan structures could be characterized by lectin blots and ultrasensitive mass spectrometer. These will give the precise glycosylation sites and glycan structures on DEFB126.

The impaired glycocalyx is associated with the reduced sperm fertility^4^,^43^. DEFB126 is a highly glycosylated protein and the carbohydrates of the protein contributes substantially to the sperm glycocalyx^11^. The homozygous two-nucleotide deletion in the *DEFB126* gene causes impaired sperm function^44^. In the current study, however, the sperm with *del*/*del* presented normal semen parameters (Table 1), which suggested the routine examination in clinic could not fully assess the male fertility. In addition, qPCR, sequencing and western blotting can only identify the mutation types of genes and the quantity of proteins. But we observed that some sperm with *del*/*del* also had the DEFB126 protein by western blotting (Fig. 4b). Thus, none of these methods can demonstrate the status of protein’s post-translational modification. However, the 6 lectins, especially ABA and MPL (AUC > 0.7), could be employed to assess the quality of the sperm glycocalyx that may be defective because of the mutation in *DEFB126.* Such results suggested that these 6 lectins could have potential capability to serve as a biomarker individually or as a combination in diagnosing males with unexplained infertility due to *DEFB126* mutation, which can offer a new insight into sperm infertility, especially into unexplained infertility.

Although it provided promising biomarkers for diagnosing the unexplained infertile patients with normal semen parameters, the current study had some limitations, which are to be addressed in our future studies. The cut-off value established with the data should be validated with an independent set of samples for increasing sensitivity and specificity. Moreover, it is necessary that prospective validation of the biomarkers be executed in a large set of samples before the test is applied to the clinic.

In conclusion, to globally profile the surface glycans of human sperm and explore the changes of glycans in sperm with *del*/*del*, we pioneered in optimizing the procedures of lectin microarray for detecting human sperm surface glycome. Through the technique, the sperm of three genotypes, i.e., *wt*/*wt*, *wt*/*del* or *del*/*del* were compared on the lectin microarray, 6 lectins identified to serve as potential biomarkers for subfertility diagnosis due to *DEFB126* mutation. Our research on the defective glycocalyx of sperm provides insight into the detection of some unexplained male subfertility. In addition, the lectin microarray strategy can be employed to investigate the glycomic profiling of sperm related to glycocalyx changes in sperm capacitation, acrosome reaction, sperm-egg recognition, which will facilitate a deep insight into the glycocalyx-related functions of human sperm.

## Methods

### Sperm collection and preparation

Human semen samples were collected in Shanghai Ji Ai Genetics & IVF Institute. One hundred and twenty donors were recruited in this study. All the semen were evaluated for sperm concentration, total motility, viability and round cell concentration according to the fifth edition of WHO laboratory manual. The samples with normal semen parameters were included; i.e., they presented the normal concentration (≥15 × 10^6^/ml), total motility (≥40%), viability (≥58%) and the low round cell concentration (≤1 × 10^6^/ml). A small fraction (<300 μl) of each sample was used for *DEFB126* genotyping. From the rest, the whole semen was centrifugated (500 g × 10 min) for collecting the sperm cells and washed with PBS, and then fixed with 2% paraformaldehyde containing 0.2% glutaraldehyde for 30 min, followed by twice washes with PBS, before stored at 4 °C for the subsequent lectin microarray and flow cytometry experiments. For other tests, the preparations of semen were described at the following each section in detail. The use of semen was allowed by the donors with written informed consent. This research was approved by the Institutional Review Committee of Fudan University. All experiments were performed in accordance with the relevant guidelines and regulations.

### Preparation and analysis of Lectin microarray

Lectin microarray was prepared as previously described^27^,^32^. Ninety-one lectins were all purchased from EY Laboratories (San. Mateo, CA) and Vector Laboratories (Burlingame, CA), and other chemicals were purchased from Sigma-Aldrich (Shanghai, China). Lectin microarrays were prepared as we had reported previously^27^. The lectins were dissolved in PBS with 0.02% Tween-20, 25% glycerol and 0.05 μg/μl bovine serum albumin (BSA) at a final concentration of 1 μg/μl and then printed on OPPolymer Slide H slides (CapitalBio, Beijing, China) with SmartArray^TM^-48 microarrayer (CapitalBio, Beijing, China). Each lectin was printed on blocks in triplicate with 18 × 16 arrangement, with 12 blocks printed on one slide. Afterwards, the slides were incubated at 4 °C overnight to ensure that lectins coated the surface. The prepared slides were stored at 4 °C for the further experiments.

Lectin microarrays were washed in 10 mM Tris Buffered Saline with 0.5% (v/v) Tween-20 (TBST) for 1 h, and in PBS with 0.5% Tween-20, then in PBS twice with gentle shaking, followed by air-drying at room temperature.

The fresh sperm were collected by centrifugation and labeled with 10 μM Carboxy-Fluorescein diacetate, Succinimidyl Ester (CFSE; Invitrogen, Carlsbad, CA) for 10min at room temperature (RT), and the fixed sperm were labeled with 20 μg/ml propidium iodide (PI; Sigma-Aldrich, Shanghai, China). Each block of lectin microarray was seeded with CFSE- or PI-labeled sperm in 200 μl PBS with 50 μM CaCl_2_ and 50 μM MnCl_2_, and then incubated in a wet box for 1 h at room temperature (RT) in the dark. Each sample was repeated four times in and between slides in a diagonal manner. The excess and unbound sperm were gently removed by submerging and inverting the slides in PBST. With the scanning condition set to 532 nm filter and 40% PMT value, the air-dried slides were scanned with a GenePix 4200A (Molecular Devices, Sunnyvale, CA) at 5 μm resolution.

### Protein preparation and western blotting analysis

The liquefied semen was centrifuged to discard the seminal plasma, and the pellets were resuspended with 1 × SDS-PAGE loading buffer including β-mercaptoethanol in the ratio of 10^6^ sperm to 100 μl loading buffer before they were boiled for 5 min. The supernatant proteins were stored at −80 °C with aliquots for the western blotting. In each sample, 10 μl proteins were separated by 12.5% SDS-PAGE and semi-dry blotted to PVDF (Polyvinylidene Fluoride) membranes (Millipore, Bedford, MA, USA). Blocked 2 h within 1 × NET, the membranes were incubated with primary rabbit polyclonal antibody to DEFB126 (1:1000; Santa Cruz, California, USA) overnight at 4 °C and then incubated with the HRP-labeled secondary antibody (1:10000; Cwbiotech, Beijing, China) for 1 h at RT. The bands, when washed thrice in TBST, were detected by ECL kit (GE Amersham, Pittsburgh, USA). The semi-quantification of target protein and reference protein were analyzed by ImageJ 1.48 gray scale scanning software.

### Immunofluorescence and scanning confocal microscopy

The smears were fixed with 2% PFA containing 0.3% Tween-20 and 0.2% Triton X-100 for 30 min. The slides, washed thrice with PBS and blocked with 30% donkey serum including 2% BSA for 1 h at RT, were incubated with rabbit polyclonal antibody to DEFB126 (1:200) overnight at 4 °C, followed by an incubation with the donkey anti-rabbit IgG conjugated with Alexa Fluor 488 (1:200; Molecular Probes, California, USA) for 1 h at RT. The negative control was incubated with rabbit IgG in the same conditions as the rabbit polyclonal antibody to DEFB126. One drop of Dapi Fluoromount-G (SouthernBiotech, Birmingham, Alabama, USA) was added to the sperm slides to be air-dried for 5 min. The slides were examined under BX51 Fluorescence microscope (Olympus, Japan) and a laser confocal scanning microscope (Leica TCS SP5, Mannheim, Germany).

### Genotype analysis of *DEFB126* (rs11467417)

From the semen, the genomic DNA was extracted as a template in a real time PCR (qPCR) to identify *DEFB126* genotype. The sequences of the genotyping primers were as follows: *wt*/*wt* (169-bp) forward primer 5′-AAGGGACTGCTGTGTTCCAG-3′, reverse primer, 5′-ACCAGTGGGAGAAACGGGCGT-3′; *del*/*del* (295-bp) forward primer 5′-CTTCGATGGCTCCTACGCG-3′, reverse primer 5′-GCTGTGGGCCTAGAACTGTC-3′. The qPCR cocktail solution contained 1.5 μl genomic DNA, 0.5 μl false-paired or right-paired primers, 5 μl Sharpvue 2 × universal qPCR Master Mix (Biovue Technology, Shanghai, China), and 3 μl nuclease-free ddH_2_O. The cycling conditions were as follows: 94 °C for 10 min, followed by 94 °C for 10 s and 60 °C for 1 min for 40 cycles; consequently, the melting curves were acquired from 60 °C to 94 °C, with an increasing rate 0.1%. The loci of interest were genotyped based on the melting temperature shift genotyping assay.

### Lectin flow cytometry

The selected lectins were used to stain the fixed sperm by 2% paraformaldehyde containing 0.2% glutaraldehyde, 5 × 10^6^ sperm re-suspended in PBS and incubated with 100 μg/ml of fluorescein isothiocyanate (FITC)-labeled lectins for 30 min at 37 °C in the dark. The sperm were washed and re-suspended with 500 μl PBS, to be analyzed in a Facs Calibur Flow cytometer using WinMID2.9 software.

### Statistical analysis

The binding signals of the sperm were extracted via GenePix pro 6.0 from the lectin microarray images. The signal intensity to the local background noise ratio (SNR) was defined as F532 Mean/B532 Mean, and all the spots’ SNRs of lectin microarray were calculated and normalized. SNRs of the 12 replicate spots, four blocks repeated with triplicate spots on each block, were averaged for each lectin. The data of the sperm of different genotypes (*wt*/*wt*, *wt*/*del* or *del*/*del*) that bound with lectins was classified and averaged, respectively. The cut off of the positive lectin binding was set as SNR ≥ 2.

Data analysis and graphs were conducted by SPSS16.0 and GraphPad Prism 5 and all the data were described as the mean ± SEM. The significant differences of the SNRs and the Geo Mean among samples with *wt*/*wt*, *wt*/*del* or *del*/*del* genotype were determined by One-way analysis of variance (one-way ANOVA). Differences were considered as significant at *p* < 0.05. ROC analyses were performed with the ABA/MPL binding signal intensity plotted against *del*/*del*. The area under the ROC curves (AUC) were calculated to evaluate the subfertility of sperm.

## Additional Information

**How to cite this article**: Xin, A. *et al.* Lectin binding of human sperm associates with *DEFB126* mutation and serves as a potential biomarker for subfertility. *Sci. Rep.* **6**, 20249; doi: 10.1038/srep20249 (2016).

## Supplementary Material

Supplementary Information

## Figures and Tables

**Table 1:** Association between the three *DEFB126* genotypes and the general semen parameters.

	*wt*/*wt*(n = 30)	*wt*/*del*(n = 30)	*del*/*del*(n = 30)	*P*
Sperm concentration (×10^6^/ml)	54.60 ± 3.15	59.05 ± 4.18	59.27 ± 3.80	0.60
Total motility (%)	39.97 ± 2.29	43.42 ± 2.46	42.71 ± 2.51	0.57
Sperm viability (%)	58.41 ± 2.64	62.25 ± 2.63	63.33 ± 2.37	0.35
Round cell concentration (×10^6^/ml)	0.58 ± 0.05	0.52 ± 0.04	0.59 ± 0.05	0.48

All values are means ± SEM.
