# Supplementary material for: Lectin binding of human sperm associates with DEFB126 mutation and serves as a potential biomarker for subfertility
Source: Sci Rep. 2016 Feb 1;6:20249. doi: 10.1038/srep20249 (PMC4735291; doi:10.1038/srep20249)
Supplement: Supplementary Information [file srep20249-s1.doc]

**Lectin binding of human sperm associates with *DEFB126* mutation and serves as a potential biomarker for subfertility**

Aijie Xin, Li Cheng, Hua Diao, Yancheng Wu, Shumin Zhou, Changgen Shi, Yangyang Sun, Peng Wang, Shiwei Duan, Jufen Zheng, Bin Wu, Yao Yuan, Yihua Gu, Guowu Chen, Xiaoxi Sun, Huijuan Shi, Shengce Tao, Yonglian Zhang

**Supplementary Figure S1. The optimization of sperm numbers for lectin-sperm binding by lectin microarray.** The sperm binding intensity with lectin mciroarray increased with sperm numbers.

**
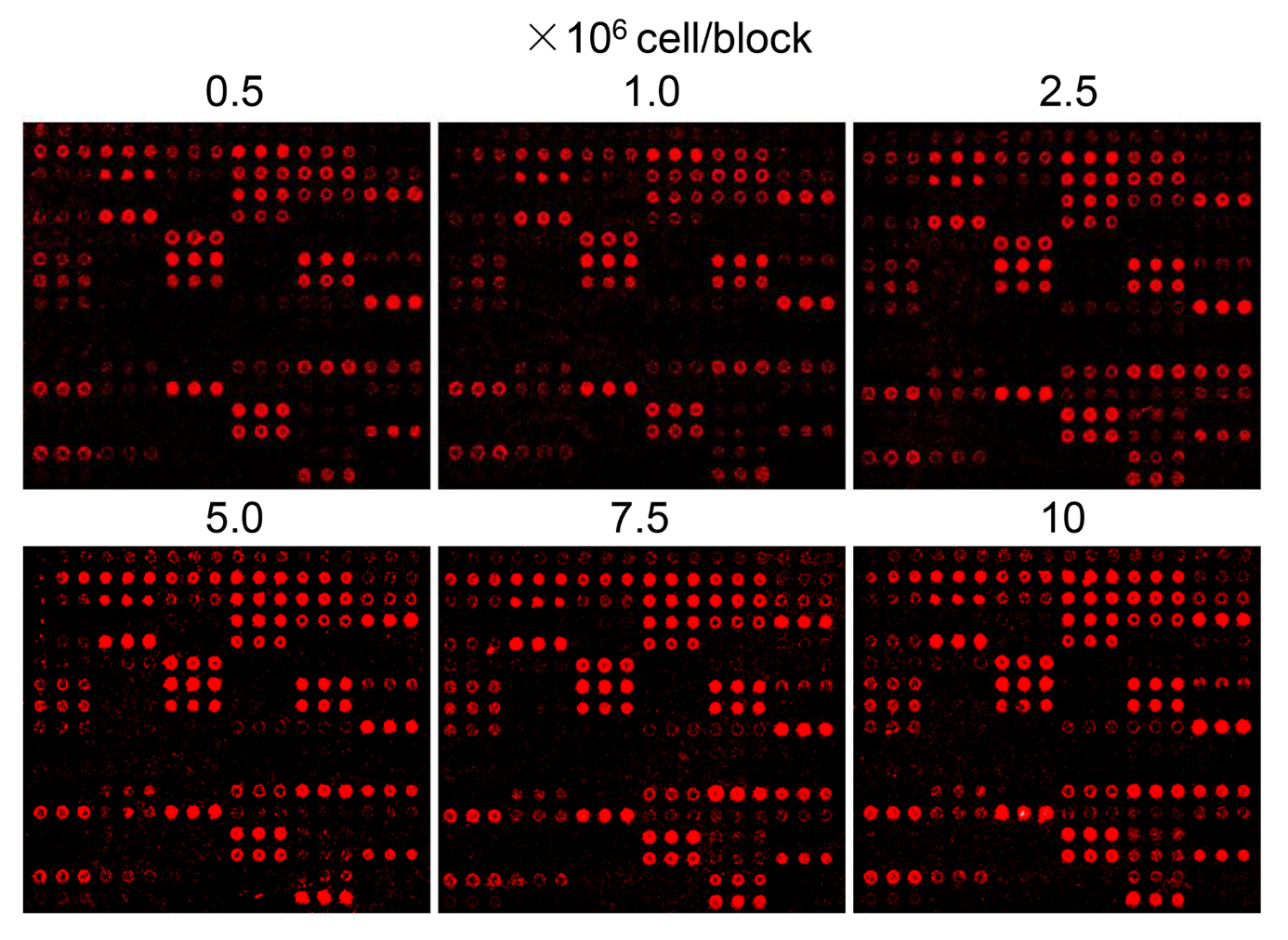
**

**Supplementary Figure S2. The effect of storage time on the binding intensity of the fixed sperm.** (**a**) The average SNRs of all the lectins on the lectin microarray after the sperm was fixed and stored for 0-6 months. (**b**) The sperm-lectin binding intensity of the five representative lectins.

**Supplementary Figure S3. Reproducibility of the lectin microarray for sperm analysis.** The average SNRs of the total lectins showing no significant difference between the two blocks; the corresponding signal images shown in right panel.


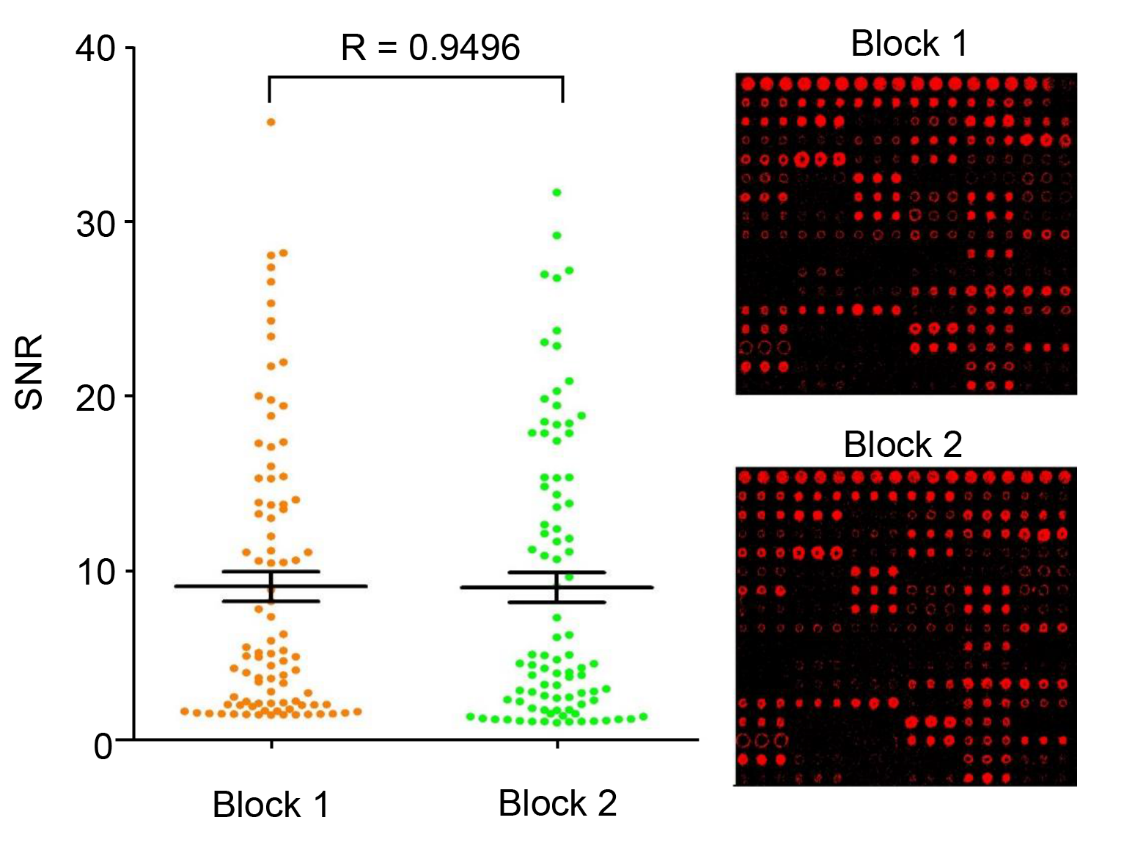


**Supplementary Figure S4. Localization of DEFB126 (green) on human sperm with different genotypes (*wt*/*wt*, *wt*/*del* and *del*/*del*) by confocal microscope.** The sperm were stained with the DEFB126 specific antibody followed by a fluorescent second antibody. The nuclei of the sperm were shown by DAPI (blue) staining.


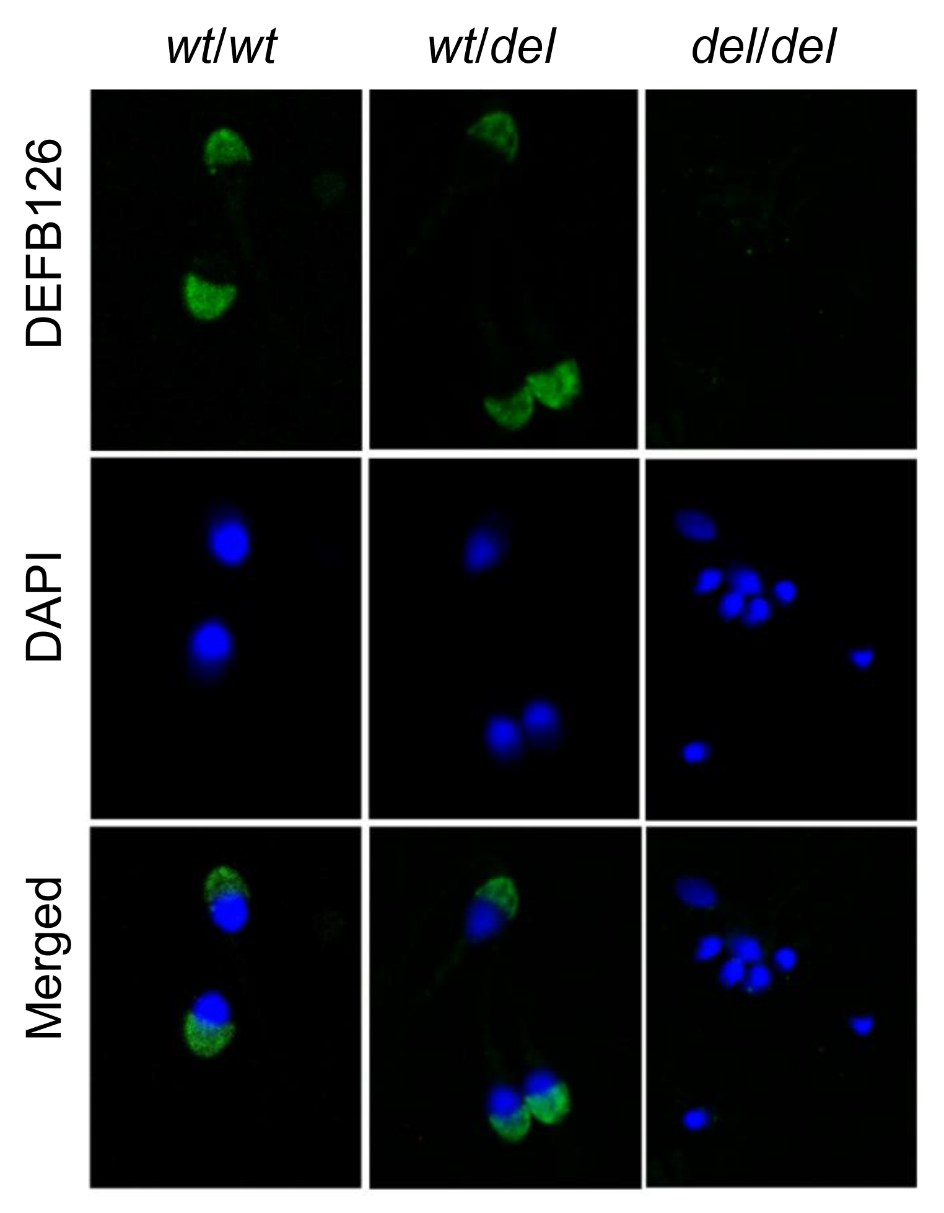


**Supplementary Figure S5. The average SNRs of PSA** **among human sperm with *wt*/*wt*, *wt*/*del* and *del*/*del* by lectin microarray.** PSA binding signal showing no signifiant difference (*P* = 0.15) among human sperm with different genotype ($\overline{\text{x}}\pm\text{SEM}$; 10 samples of each genotype).


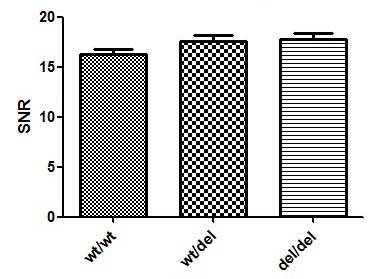


**Supplementary Table S1.** **Association between the three *DEFB126* genotypes and the general semen parameters of sperm used for lectin microarray.**

|  | *wt*/*wt*  (n = 10) | *wt*/*del*  (n = 10) | *del*/*del*  (n = 10) | *P* |
| --- | --- | --- | --- | --- |
| Sperm concentration (× 10^6^/ml) | 53.16 ± 8.42 | 62.16 ± 2.92 | 57.46 ± 8.49 | 0.67 |
| Total motility (%) | 48.19 ±3.07 | 51.24 ± 2.48 | 52.93 ± 2.36 | 0.46 |
| Sperm viability (%) | 64.14 ± 3.90 | 65.43 ± 4.40 | 69.29 ± 3.69 | 0.65 |
| Round cell concentration (× 10^6^/ml) | 0.49 ± 0.10 | 0.46 ± 0.07 | 0.56 ± 0.10 | 0.74 |

All values are means ± SEM.
